# Phylogenetic and Phylogenomic Definition of *Rhizopus* Species

**DOI:** 10.1534/g3.118.200235

**Published:** 2018-04-19

**Authors:** Andrii P. Gryganskyi, Jacob Golan, Somayeh Dolatabadi, Stephen Mondo, Sofia Robb, Alexander Idnurm, Anna Muszewska, Kamil Steczkiewicz, Sawyer Masonjones, Hui-Ling Liao, Michael T. Gajdeczka, Felicia Anike, Antonina Vuek, Iryna M. Anishchenko, Kerstin Voigt, G. Sybren de Hoog, Matthew E. Smith, Joseph Heitman, Rytas Vilgalys, Jason E. Stajich

**Affiliations:** * Department of Biology, Duke University, Durham, North Carolina, 27708; † Department of Botany and Department of Bacteriology, University of Wisconsin-Madison, Madison, Wisconsin, 53706; ‡ Westerdijk Fungal Biodiversity Institute, Utrecht, The Netherlands, 3584; § US Department of Energy, Joint Genome Institute, Walnut Creek California, 94598; ** Department of Microbiology & Plant Pathology and Institute for Integrative Genome Biology, University of California Riverside, Riverside, California, 92521; †† School of BioSciences, University of Melbourne, Melbourne, Victoria, Australia, 3010; ‡‡ Institute of Biochemistry and Biophysics, Polish Academy of Sciences, Warsaw, Poland, 02-106; §§ Laboratory of Bioinformatics and Systems Biology, Centre of New Technologies, University of Warsaw, Warsaw, Poland, 02-089; *** North Florida Research and Educational Center, University of Florida, Quincy, Florida, 32351; ††† Department of Natural Resources and Environmental Design, North Carolina Agricultural and Technical State University, Greensboro, North Carolina, 27401; ‡‡‡ Department of Plant Protection, National University of Life and Environmental Sciences of Ukraine, Kyiv, Ukraine, 03041; §§§ M.G. Kholodny Institute of Botany, National Academy of Sciences of Ukraine, Kyiv, Ukraine, 02000; **** Leibniz Institute for Natural Product Research and Infection Biology, Hans-Knöll Institute, Jena, Germany, 07745; †††† Department of Plant Pathology, University of Florida, Gainesville, Florida, 32611; ‡‡‡‡ Department of Molecular Genetics and Microbiology, Duke University Medical Center, Durham, North Carolina, 27710; §§§§ Faculty of Engineering, Sabzevar University of New Technologies, Sabzevar, Iran

**Keywords:** zygomycete, orthologs, genome duplication, transposons, sexual reproduction

## Abstract

Phylogenomic approaches have the potential to improve confidence about the inter-relationships of species in the order Mucorales within the fungal tree of life. *Rhizopus* species are especially important as plant and animal pathogens and bioindustrial fermenters for food and metabolite production. A dataset of 192 orthologous genes was used to construct a phylogenetic tree of 21 *Rhizopus* strains, classified into four species isolated from habitats of industrial, medical and environmental importance. The phylogeny indicates that the genus *Rhizopus* consists of three major clades, with *R. microsporus* as the basal species and the sister lineage to *R. stolonifer* and two closely related species *R. arrhizus* and *R. delemar*. A comparative analysis of the mating type locus across *Rhizopus* reveals that its structure is flexible even between different species in the same genus, but shows similarities between *Rhizopus* and other mucoralean fungi. The topology of single-gene phylogenies built for two genes involved in mating is similar to the phylogenomic tree. Comparison of the total length of the genome assemblies showed that genome size varies by as much as threefold within a species and is driven by changes in transposable element copy numbers and genome duplications.

*Rhizopus* (Ehrenb. 1821) is a genus of saprotrophic zygomycete fungi (Mucoromycotina, Mucoromycota) that is ubiquitous in soil, animal excrement, and rotting vegetation (Pidoplichko and Mil’ko 1971). The genus is especially relevant to human enterprises. For example, certain species can act as plant pathogens that affect crops, some are producers of enzymes in industrial biofermentation, and others are used as fermentation agents in food production. Furthermore, certain species are causal agents of disease in animals, including humans, and are used as model organisms in the study of fungal cellular and molecular biology (Abe *et al.* 2006, Ogawa *et al.* 2004, Saito *et al.* 2004, Muszewska *et al.* 2014).

Some *Rhizopus* species present a significant threat to post-harvest agricultural products by damaging the appearance and taste of crops, most notably sweet potatoes and strawberries (Eckert 1978, Tournas 2005). Infection can also lead to human poisoning due to release of the phytotoxin rhizoxin, which is synthesized by endosymbiotic *Burkholderia* bacteria inhabiting the hyphae of some *Rhizopus* species (Partida-Martinez *et al.* 2007). *Rhizopus* is also an opportunistic agent of human and animal disease in immunocompromised individuals and causes approximately 60–80% of all disease manifestations of mucormycosis (Ibrahim *et al.* 2008, Ma *et al.* 2009). Although *Rhizopus*-associated mucormycosis is less common than fungal infections caused by ascomycete species (*e.g.*, *Candida* or *Aspergillus*) or basidiomycete species (*e.g.*, *Cryptococcus*), mucormycosis has an overall mortality rate exceeding 50%, and the number of cases with fatal outcomes is currently increasing, especially in patients with combat-related injuries or vascular invasion (Muszewska *et al.* 2014, Tribble and Rodriguez 2014).

For centuries, *Rhizopus* species have been used in the production of fermented products such as tempeh and ragi (Ogawa *et al.* 2004, Dolatabadi *et al.* 2016). More recently, *Rhizopus* species have proved useful in bioindustrial pursuits to synthesize metabolites. For example, species of the *R. arrhizus/delemar* complex are used to produce lactic, fumaric, malic, and other organic acids, as well as in the synthesis of ethanol, carotenoids, and some hydrolytic enzymes (Abe *et al.* 2003).

Given the importance of *Rhizopus* in both human health and industry, a robust classification system is needed to reflect the key differences between species and how the relationships between species correlate with properties related to human activities. Species traditionally have been differentiated based on discrete morphological and physiological features, such as the maximum growth temperature, formation of morphological structures (chlamydospores, sporangia, and rhizoids), curvature of the columella, sporangiophore features (color, shape, and size), sporangia diameter, acid production, and results of the Voges-Proskauer test (a test of acetoin production) (Inui *et al.* 1965). A karyological study of *Rhizopus* strains isolated from Korean soil showed that chromosome number can vary from a minimum of 8 in *R. delemar* and *R. arrhizus* to a maximum of 16 in *R. stolonifer* (Min 1984). The *Rhizopus* classification published by Schipper (Schipper 1984) separated the genus into three groups—*R. microsporus*, *R. stolonifer*, and *R. arrhizus* (=*oryzae*)—based on rhizoid branching, growth temperature and the size of sporangia and sporangiophores. In 2006, Abe *et al.* (Abe *et al.* 2006) confirmed the same taxonomic grouping in the first molecular phylogenetic study of the genus. In 2007 Liu *et al.* (Liu *et al.* 2007) organized the genus into 10 species and seven varieties using ribosomal DNA (rDNA) and orotidine-5′-monophosphate decarboxylase (*pyrG*) sequences. In the same year, Zheng *et al.* (Zheng *et al.* 2007) reanalyzed the data from Liu *et al.* (Liu *et al.* 2007) along with morphological data, and they instead divided the genus into eight species. Due to uncertainties in the phylogenetic analyses (specifically concerning the placement of *R. americanus* syn. *R. stolonifer*), Abe *et al.* (Abe *et al.* 2010) in 2010 used rDNA ITS, actin-1, and translation elongation factor 1α (*EF-1α*) sequences to confirm the eight-species division of *Rhizopus*. The fungal species database Index Fungorum (http://www.indexfungorum.org) identifies 11 *Rhizopus* species, whereas zygomycetes.org (http://zygomycetes.org/index) lists 13, 11 of which might be valid taxonomic names and represent *bona fide* species. However, most *Rhizopus* samples in culture collections belong to four species or species complexes: *R. microsporus*, *R. stolonifer*, *R. arrhizus* (or *R. oryzae*), and *R. delemar* (or *R. arrhizus* var. *delemar*). Other *Rhizopus* species are rarely collected or deposited in culture collections and lack representation within sequence databases (Table 1). These rare species were thus the first targets for whole-genome sequencing to better understand their environmental, medical, and biotechnological applications. Except for species chosen for genome sequencing projects, only a handful of genes or DNA regions have been sequenced in other *Rhizopus* species. Therefore, few known variable nucleotide sites are available to resolve relationships between *Rhizopus* species, and published phylogenies of single or multiple genes differ in topology, even with the inclusion of the same genes or gene regions (Liou *et al.* 2007, Liu *et al.* 2007, Abe *et al.* 2010, Hoffmann *et al.* 2013). Single-gene phylogenies can be inconsistent with the species phylogeny due to insufficient or conflicting phylogenetic signals caused by non-uniform rates of molecular evolution or genetic exchange among lineages. Inferences of species phylogenies from one gene, or a few genes, assume that each gene shares the same evolutionary history as the whole organism, an assumption that is not consistently supported (Fitzpatrick *et al.* 2006). Sampling a larger number of genes permits the resolution of the phylogenetic relationship as well as analysis of conflict among individual genes.

**Table 1:** Census of *Rhizopus* taxa in three major culture collections and the NCBI databases (as of May 5, 2017). The four species with the greatest number of identified isolates are shown in bold

Species	ATCC	Westerdijk Institute (CBS-KNAW)	CABI	GenBank records*^a^*	PubMed records
***R. arrhizus***	**137**	**76*^b^***	**39*^b^***	**7,451*^b^***	**2133*^b^***
*R. caespitosus*	—	1	—	14	15
*R. circinans*	7	—	—	12	11
***R. delemar***	**-*^c^***	**12**	**-**	**2,824**	**155**
*R. homothallicus*	2	2	6	23	34
*R. lyococcus*	—	3	—	8	4
***R. microsporus**^d^***	**70**	**48**	**29**	**3,645**	**527**
*R. niveus*	1	—	—	72	127
*R. schipperae*	2	1	—	27	14
*R. sexualis*	3	3	4	39	19
***R. stolonifer***	**30**	**18*^e^***	**14**	**299**	**413**
*Rhizopus* sp.*^f^*	1	3	—	269	4182

a - Including all genes.

b - Including *R. arrhizus* and *R. arrhizus var. delemar*.

c - Together with *R. arrhizus*.

d - Including *R. azygosporus* and *R. oligosporus*.

e - Including *R. stolonifer var. reflexus*.

f – Not identified to the species level.

The main goal of this study was to elucidate major evolutionary trajectories in *Rhizopus* using previously published whole-genome sequences supplemented with additional new genomes generated in this project. We have produced a genus-level phylogeny of four species using phylogenomic approaches and compared the topology to single-gene phylogenies of genes that are important in the *Rhizopus* reproductive cycle. We compared our consensus species tree to the gene trees of RNA helicase (*rnhA*) (Calo *et al.* 2017), a gene adjacent to the *sex* mating locus, and the 4-dihydrotrisporin-dehydrogenase (*tsp2*) gene that is involved in the synthesis of trisporic acid, a trigger of the mating process in mucoralean fungi (Wetzel *et al.* 2009). We also assessed the contribution of transposable elements and genome duplication to the variance in genome size across the genus, as has previously been deduced for *R. delemar* and other mucoralean fungi (Ma *et al.* 2009, Corrochano *et al.* 2016). We also directly compared our phylogenetic results with a morphological phylogeny of the genus and found that they are congruent.

## Materials and methods

### Genome sequencing, assembly, and annotation

Cultures of *R. azygosporus* strain CBS 357.93 and *R. stolonifer* strain LSU 92-RS-3 were grown on 1% potato dextrose agar (PDA, NEOGEN, Lansing, MI, USA). Three 0.5×0.5-cm pieces were cut from the edge of 5-day-old colonies and homogenized in a Waring Blender for further use as inoculum in liquid potato dextrose broth (0.5%). Cultures were grown in 250-mL Erlenmeyer flasks in 50 mL of medium on a shaker at room temperature for five days in three replicates. Before harvesting, the samples were examined microscopically to confirm the absence of bacterial or fungal contamination. Consolidated tissue was filtered through sterilized Miracloth (Skory and Ibrahim 2007) and washed twice in sterile distilled water before DNA extraction. The mycelial biomass was then lyophilized for one to two days and ground in liquid nitrogen with a mortar, pestle, and sterilized sand. DNA was extracted with 2× CTAB buffer following a modified DNA chloroform extraction technique (Gardes and Bruns 1993). To prevent nucleic acid degradation, the samples were not incubated in a water bath prior to the addition of chloroform. The sample quality was verified by SYBR Safe staining on 0.8% agarose gels to detect nucleic acid contamination and traces of degradation. The total quantity of high molecular weight DNA was estimated using Quantity One 1-D analysis software with a Gel Doc UV transilluminator (Bio-Rad, Hercules, CA, USA). Genomic DNA was sequenced in 2×100 paired-end reads on Illumina HiSequation 2000 at the High-Throughput Genomic Sequencing Facility of the University of North Carolina, Chapel Hill, NC, USA, and assembled using Celera v. 8.2. The analysis also incorporated the high-quality assembly of *R. delemar* strain RA 99-880, the first published genome of a Mucorales species (Table 2, Ma *et al.* 2009).

**Table 2:** Origin of the genome data

Species	Collection and strain	BioProject	Size, Mb	Gene number	GC%	Sequencing method	Coverage	Assembly	Isolated from
*Rhizopus arrhizus*									
	NRRL 13440	PRJNA186013	43.351	11,871	35.2	Illumina HiSeq	86.09×	MaSuRCA v.1.9.2	tracheal biopsy
	NRRL 18148	PRJNA186014	47.535	12,599	35.0	-//-	22.51×	-//-	sinus
	NRRL 21396	PRJNA186017	42.783	11,715	35.2	-//-	64.45×	-//-	sinus
	UCLA 99-113	PRJNA186016	41.453	11,995	35.4	-//-	18.30×	-//-	bone marrow
	UCLA 99-892	PRJNA186020	37.464	11,675	35.2	-//-	85.45×	Velvet v.1.2.07	lung transplant
(*Mucor ramosissimus*^a^)	UCLA 97-1182	PRJNA186024	42.900	12,951	35.3	-//-	73.11×	MaSuRCA v.1.9.2	bronchial wash
	CDC B7407	PRJNA184879	43.272	11,664	34.9	-//-	47.77×	-//-	nasal cavity
	UCLA HUMC 02	PRJNA186018	39.011	11,785	34.6	-//-	103.09×	Velvet v.1.2.07	sinus
*R. delemar*									
	NRRL 21446	PRJNA186022	36.999	11,402	35.5	-//-	75.13×	-//-	face biopsy
	NRRL 21447	PRJNA186021	37.254	11,387	35.5	-//-	80.49×	-//-	brain, ear
	NRRL 21477	PRJNA186019	38.882	11,523	34.8	-//-	80.75×	-//-	face biopsy
	NRRL 21789 *(R. oryzae*^b^)	PRJNA186015	42.018	11,414	35.4	-//-	41.53×	MaSuRCA v.1.9.2	sinus
	UCLA 99-880 (*R. oryzae*^b^)	PRJNA13066	45.263	12,384 (17,467)	35.6	Sanger ABI	14.00×	Arachne v.1.0	brain abscess
*R. microsporus*									
(*R. azygosporus*^c^)	CBS-KNAW 357.93	PRJNA418064	15.920	4,430	36.8	Illumina HiSeq	unknown	Celera v.8.2	tempeh
	ATCC 52813	PRJNA205957	25.348	8,847 (10,905)	37.5	-//-	143.6×	AllPathsLG v. R41043	soil
	CDC B9738	PRJNA211903	75.133	21,091	33.3	-//-	37.36×	MaSuRCA v.1.9.2	abdomen
	CCTCC M201021	PRJNA179339	45.700	15,773 (20,087)	36.9	-//-	100.00×	SOAPdenovo v.1.12	liquor leaven
	CDC B7455	PRJNA211913	48.730	16,729	37.2	-//-	37.36×	-//-	abdomen
(*M.racemosus*^d^)	UMSoM B9645	PRJNA211902	65.533	17,671	32.5	-//-	49.38×	-//-	floor
*R. stolonifer*									
	LSU 92-RS-03	PRJNA418064	29.733	11,621	**37**	-//-	unknown	Celera v.8.2.	sweet potato
	CDC B9770	PRJNA184886	38.026	11,778	35.5	-//-	42.23×	MaSuRCA v.1.9.2	contaminated product
*M. circinelloides* (outgroup)									
	DUSoM 1006PhL	PRJNA172437	34.135	12,693	39.5	-//-	45.00×	ALLPATHS v.R43527	unknown
	CDC B8987	PRJNA184880	36.701	13,407	39.5	-//-	100.43×	-//-	BL line

Incorrectly identified strains are shown in parentheses: a - *R. arrhizus*, b - *R. delemar*, c and d - *R. microsporus*.

-//- = same as above.

Culture collections: ATCC – American Type Culture Collection; CBS-KNAW - The Centraalbureau voor Schimmelcultures, Westerdijk Fungal Biodiversiry Centre at institute of the Royal Netherlands Academy of Arts and Sciences; CCTCC - China Center for Type *Culture Collection*; CDC - Center for Disease Control and Prevention; DUSoM – Duke University, School of Medicine, LSU – Louisiana State University; NRRL - Northern Regional Research Lab, ARS Culture Collection of USDA; UCLA – University of California, Los Angeles; UMSoM - University of Maryland, School of Medicine.

Genome annotation was performed with MAKER (v. 2.31.8) (Holt and Yandell 2011) Augustus (2.7), SNAP (v. 2013-11-29) (Korf 2004), and GenemarkHMM (4.32) training. When available, we also considered mRNA and protein evidence using sequences from either the target species or closely related species (within the *Rhizopus* clade). The *Rhizopus oryzae* model was used for organisms on which Augustus had not been previously trained. SNAP was retrained using the results from the first run of MAKER and used to improve the gene models for a second round of annotation from the retrained prediction parameters following the best practices for the MAKER annotation protocol. Repeat masking was performed using Repeat Masker (4-0-5) through the MAKER pipeline, using fungi as the model organism. Analyses were run using the High-Performance Computing Cluster in the Institute for Integrative Genome Biology at the University of California, Riverside, CA.

### Taxon sampling

A total of 21 *Rhizopus* genomes were obtained from GenBank and the Joint Genome Institute (JGI) to represent the three major *Rhizopus* lineages: *microsporus*, *arrhizus* (*= oryzae/delemar*), and *stolonifer* (Table 2). We also selected two outgroup genomes from the genus *Mucor*: *M. circinelloides* strains 1006PhL and B8987.

### Phylogenomic resolution of the fungi

Phylogenetically informative orthologous genes from a pan-Eukaryotic dataset were selected (James *et al.* 2013). In total, 192 orthologs previously identified as primarily single-copy genes across 39 eukaryotic species were aligned with TCoffee (Magis *et al.* 2014) and incorporated into Profile Hidden Markov Models (HMM) implemented in HMMER (Wheeler and Eddy 2013). Each HMM was searched against the predicted proteome from the 23 sampled species in this study. For each ortholog, the highest scoring protein sequence in each species was identified by hmmsearch with a significance cutoff of 1^−10^. A multiple sequence alignment of orthologous sequences was generated by aligning the homologous protein sequences to the marker HMM using hmmalign. These alignments were trimmed with TrimAl (Capella-Gutiérrez *et al.* 2009) with the -strictplus parameter.

Gene trees were constructed using RAxML with the ‘-f a’ fast bootstrapped tree method on the trimmed individual alignments using PROTGAMMAAUTO and 100 bootstrap replicates to assess the clade support. The alignments were concatenated into a single super matrix alignment, and the complete tree was inferred using the RAxML ‘-f a’ fast bootstrapped tree method and PROTGAMMAAUTO model and 100 bootstrap replicates.

### Comparative genomics of sexual reproduction genes

We used BLASTP to search against predicted proteomes of each *Rhizopus* genome (Altschul *et al.* 1990) for RNA helicase (*rnhA*, accession numbers), which is adjacent to the *sex* (mating type) locus (Gryganskyi *et al.* 2010), and 4-dihydrotrisporin-dehydrogenase enzyme (*tsp2*, accession AM937248), which is required for pheromone production (Wetzel *et al.* 2009). The highest scoring contigs were searched for high mobility group (HMG) domains and triose phosphate transporters (*tptA*) in close vicinity to *rnhA*. A gene cluster consisting of an HMG domain-containing gene flanked by *rnhA*, with or without *tptA*, was considered the putative *sex* locus.

The sequences of the genes found in or near the *sex* locus were aligned using MUSCLE (Edgar 2004). The alignments were visually inspected, and ambiguous regions were excluded using Mesquite v. 3.2 (Maddison and Maddison 2018). The alignments for the *rnhA* tree consisted of 235 amino acid characters. The alignment for the *tsp2* tree consisted of 138 amino acid characters for 17 *Rhizopus* species, and the *tsp2* sequence from *Mucor mucedo* (Wetzel *et al.* 2009) was used as the outgroup. Maximum likelihood (ML) for all trees was estimated using GARLI-2.0 (Bazinet *et al.* 2014). Phylogenetic support was assessed by 1,000-bootstrap analysis using PAUP* 4.0a109 (Swofford 1998).

### Phylogenetics of ecological and morphological characters

**Figure 1:**
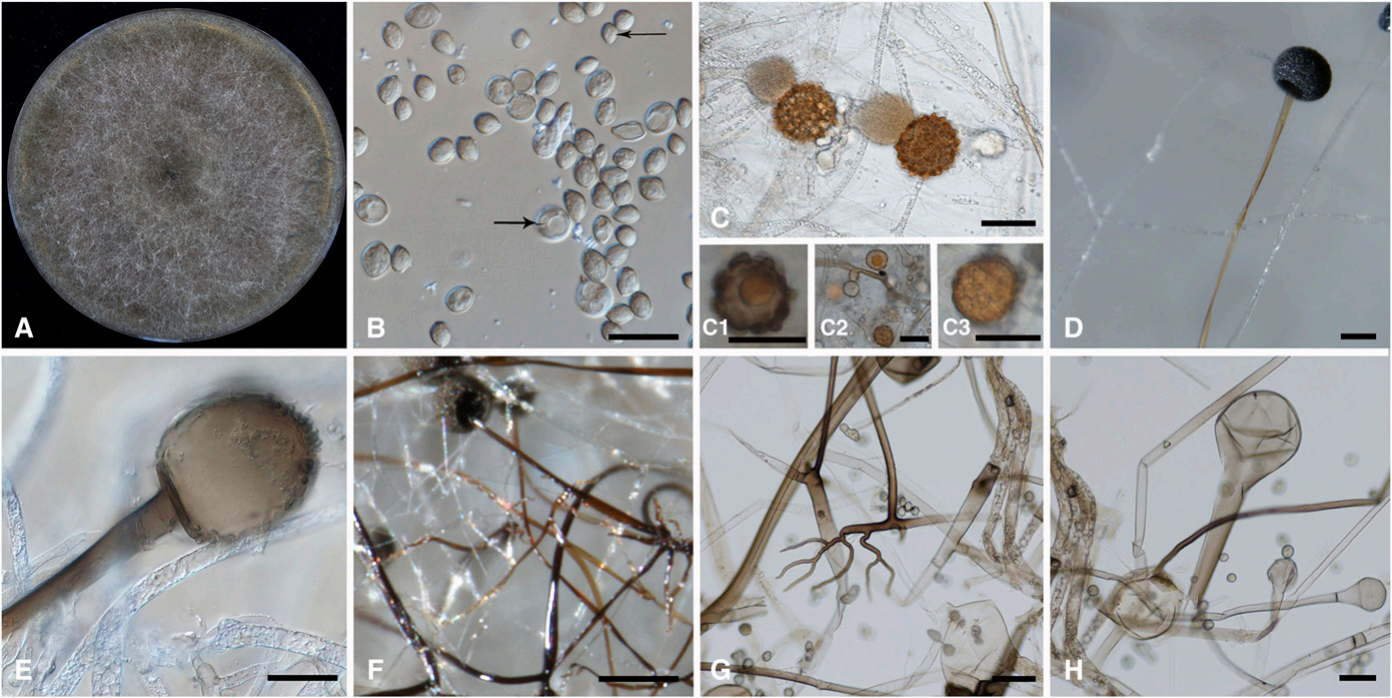
Morphology of *Rhizopus* species. (A) *R. delemar* CBS 390.34 colony on MEA after three days of cultivation at 30 °C. (B) Intact and germinating sporangiospores of *R. delemar* CBS 390.34. Arrows indicate spores of different sizes. Scale bar = 10 μm. (C) Zygospores with unequal suspensors. C1, C2, and C3 show *R. microsporus* CBS 344.29 azygospores; these were formed in the absence of a mating partner and are morphologically different from typical zygospores because they are smaller in size and have a single suspensor. Scale bar = 10 μm. (D) *R. microsporus* CBS 700.68 sporangiophore with columella. Scale bar = 10 μm. (E) *R. arrhizus* var. *arrhizus* CBS 330.53 sporangiospore release and columella. Scale bar = 10 μm. (F) Sporangiophore, rhizoids, and pigmented hyphae of *R. arrhizus* var. *arrhizus* CBS 330.53. Scale bar = 10 μm, (G) *R. stolonifer* CBS 926.87 stolons. Scale bar = 50 μm. (H) *R. stolonifer* CBS 926.87 empty sporangiophore. Scale bar = 50 μm.

We selected 16 non-molecular characters to generate a data matrix for phylogenetic reconstructions and to assess the morphological similarities between the main *Rhizopus* clades. Non-molecular data were collected directly from pertinent literature (Pidoplichko and Mil’ko 1971, Schipper 1984, Benny *et al.* 2001, Zheng *et al.* 2007, Jennessen *et al.* 2008), as well as from our own microscopic observations of cultures of *R. arrhizus*, *R. delemar*, *R. microsporus*, and *R. stolonifer* (Figure 1). Sporangia were isolated from five- to seven-day-old colonies cultured on 1% malt extract agar (MEA, Sigma-Aldrich, St. Louis, MO, USA), and observed with 10× to 40× objective lenses on an Olympus BH-2 microscope. Micromorphological features of the sporangia, sporangiospores, and sporangiophores, as well as the presence or absence of zygospores, rhizoids, and stolons, were taken into account. Additionally, we included two ecological characters (growth temperature and substrate), which are also considered to be important for the taxonomy of the genus (Table S2).

The phylogeny of the morphological characters was constructed using maximum parsimony (MP) in PAUP* 4.0a146 (Swofford 1998) with 1,000 bootstrap iterations with 10 random additions per replicate as a criterion for clade robustness.

### Transposon analysis

Transposable elements (TEs) were identified and annotated using *de novo* and homology-based approaches. Candidate TEs were identified *de novo* with the inverted repeat finding tool irf (Doerks *et al.* 2002) and RepeatModeler (Jurka *et al.* 2005). These sequences were clustered with cd-hit and scanned for protein domains related to transposons using the PFAM and CDD protein domains at pfamscan.pl, with HMMer wrapper and RPSTBLASTN+ v. 2.4.0+. TE candidates with coding regions that are similar to proteins related to transposon proteins were used. These were merged with RepBase and used as a reference in RepeatMasker (Smit *et al.* 2015). The RepeatMasker output was checked and corrected with in-house scripts, and only hits with scores higher than 200 were considered. Two datasets were generated for each genome: one with all TEs with RepeatMasker scores higher than 200 and the other with TEs that also retained similarity to typical TE-encoded protein domains.

### Data availability

*De novo* genome assembly, annotation, and raw sequence reads of the *R. azygosporus* and *R. stolonifer* are available in the NCBI Genome and SRA database linked to BioProject accession number PRJNA418064 and as accession numbers PJQM00000000 and PJQL00000000. Accession numbers of genome sequence and assembly of *Rhizopus* sp. strains from other studies utilized in this study are listed in the Table 2. Morphological and physiological data for non-molecular phylogenic analyses and their encoding are in Table S1. The types of detected transposable elements, their analysis with domains, summary and original figures are in Table S2. Sequence and structure of the *sex gene* loci are deposited in NCBI Nucleotide database under the accession numbers HQ450311-12, HQ450315-16 (*R. arrhizus*), HQ450313 (*R. delemar*), MG967658 (*R. stolonifer*), MG967659-60 (*R. microsporus* var. *azygosporus*). Sequences of single copy genes for RNA helicase and 4-dihydrotrisporin-dehydrogenase enzyme are deposited in NCBI Nucleotide database under accession numbers MG97275-98 and MG97299-324. Supplemental material available at Figshare: https://doi.org/10.25387/g3.5971426.

## Results

### Whole-genome sequencing

Genome assemblies of *Rhizopus* strains were produced with sequencing depths ranging from 10× to 144×. The assembled genome sizes varied threefold in the five *R. microsporus* strains, from 25.348 Mb in ATCC 52813 strain to 75.133 Mb in CDC B9738 strain. In the remaining *Rhizopus* strains, the assembly size was an average of 40 Mb, ranging from 29.733 Mb to 38.026 Mb in *R. stolonifer*, 37.254 Mb to 45.263 Mb in *R. delemar*, and 37.464 Mb to 47.535 Mb in *R. arrhizus*. Due to the genome size in *R. microsporus* strains the gene number varied widely, from 8,847 in ATCC 52813 strain to 17,671 in UMSoM B9645 strain, with an average 16,010 genes over all five strains tested. In other *Rhizopus* species the gene counts ranged from 11,387 to 12,951. The GC content was similar among species and varied from 32.5 to 37.5% (36% on average). The technology and quality of the sequencing data significantly influenced the quality and number of predicted genes. For example, lower quality sequencing results yielded assemblies with only 4,430 predicted genes in *R. azygosporus* CBS 357.93, which is less than half the number of genes recovered from other assemblies in the species complex.

### New phylogeny of the main lineages in the genus Rhizopus

**Figure 2:**
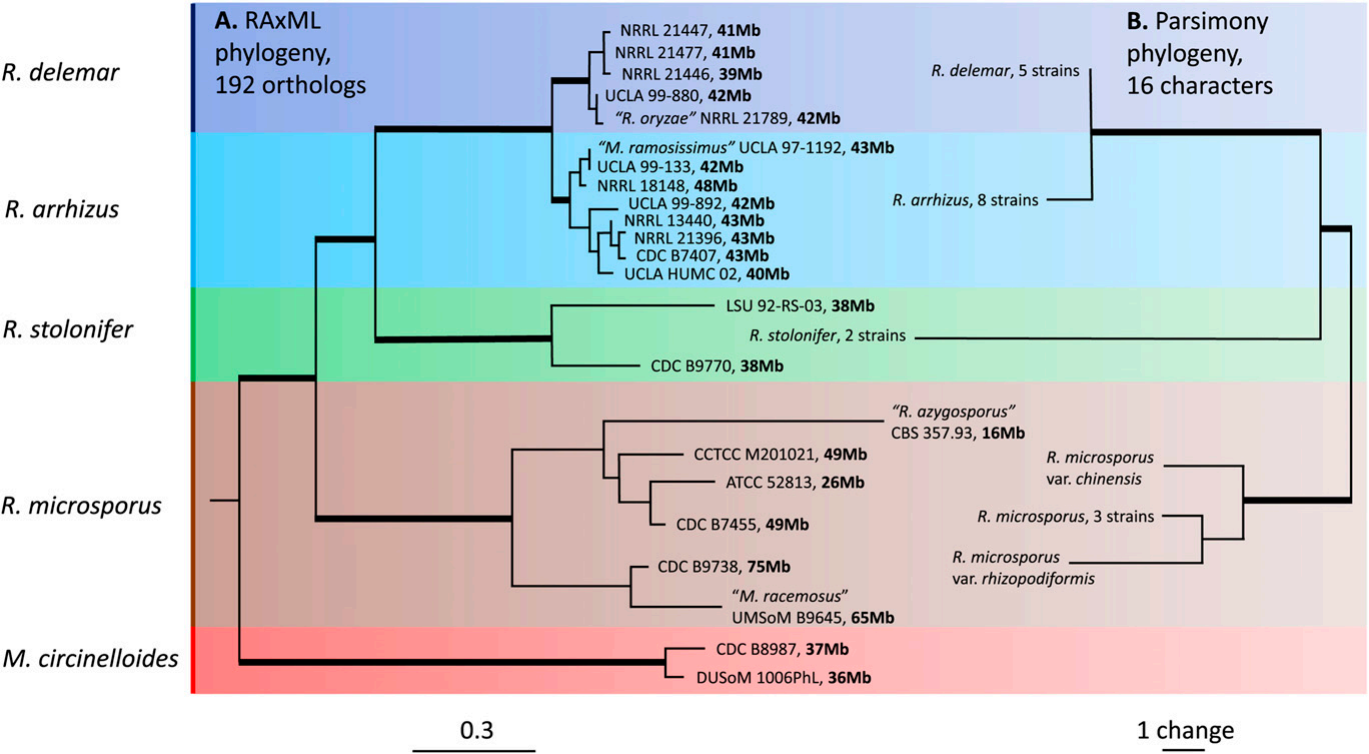
Genome-based maximum likelihood phylogeny and parsimony phylogeny based on non-molecular characters. (A) Rooted maximum likelihood tree of the genus *Rhizopus* based on 192 orthologous genes. Misidentified strains are indicated in quotes: “*Mucor racemosus*” B9645 = *R. microsporus* B9645 and “*Mucor ramosissimus*” 97-1192 = *R. arrhizus* 97-1192. Genome size is indicated in bold after the strain name. (B) Unrooted parsimony tree of 16 non-molecular (14 micromorphological and two ecological) characters. Morphological and physiological data for different strains of the same species are consolidated in the tree except for those strains that differ in at least one character. Thick branches denote statistically significant bootstrap values.

The genus *Rhizopus* is a well-defined monophyletic group that is distinct from other genera of Mucorales (Figure 2A, (Spatafora *et al.* 2016)). There are four major species or species complexes in this genus: the *microsporus*, *stolonifer*, *arrhizus*, and *delemar* clades. All of these lineages are distinct and represent reciprocally monophyletic clades with significant statistical support.

The *R. microsporus* clade is sister to the other members of the genus, and the genomes of the *R. microsporus* varieties (var. *chinensis* and var. *rhizopodiformis*) are grouped among other isolates of this species, further supporting their subspecies rank. The *R. stolonifer* strains are sister to a clade of two closely related species *R. arrhizus* and *R. delemar*. This phylogenetic tree enables corrections of some species misidentifications, namely *M. racemosus* B9645 (which is correctly identified as *R. microsporus*) and *M. ramosissimus* strain NRRL 97-1192 (which is correctly identified as *R. arrhizus*). In addition, strain NRRL 21789 (which was previously mistakenly identified as *R. oryzae* (Gryganskyi *et al.* 2010) is actually a strain of *R. delemar*. As expected, *R. azygosporus* CBS 357.93 is part of the *R. microsporus* clade, as demonstrated for other *R. azygosporus* strains by Abe *et al.* (Abe *et al.* 2006), Zheng *et al.* (Zheng *et al.* 2007), and Dolatabadi *et al.* (Dolatabadi *et al.* 2014).

We built phylogenetic trees from two single genes (*rnhA* and *tsp2*) that are likely to be important for the sexual reproduction process in these *Rhizopus* species (Wetzel *et al.* 2009, Gryganskyi *et al.* 2010, Schulz *et al.* 2016). In both phylogenies, *R. stolonifer* is included in the *R. arrhizus*/*delemar* clade but this clade is distinct from the *R. microsporus* clade. Despite the poorly resolved placement of *R. stolonifer*, the phylogeny of the *rnhA* and *tsp2* genes shares the same topology as our supermatrix tree of 192 orthologs with strong bootstrap support (Figures S1-2). Phylogenetic trees were constructed from non-molecular characters to assess the relationships among the three major clades of *Rhizopus* independently of the sequence data. The tree topologies recovered from the non-molecular parsimony analysis were congruent with the phylogenomic tree. In both analyses, *R. stolonifer* is sister to the closely related species *R. arrhizus* and *R. delemar*, whereas *R. microsporus* is sister to all of these taxa (Figure 2B).

### Genome size is highly variable, even within species

**Figure 3:**
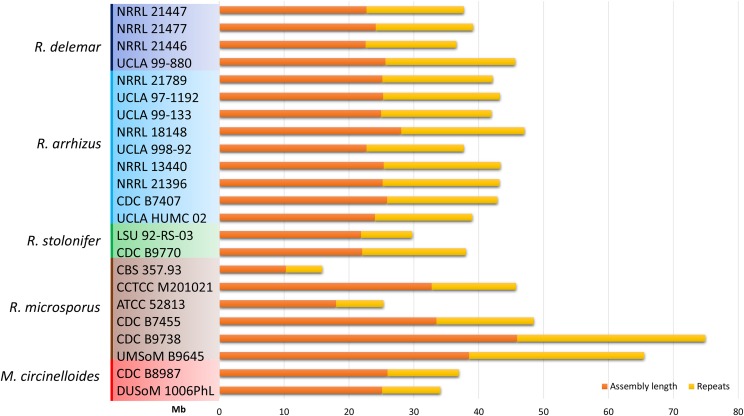
Genome size and repeat content in *Rhizopus* genomes. Colored boxes are used to highlight the species identity of each strain.

Genome size varied widely among the examined *Rhizopus* genomes. *R. microsporus* genomes are the smallest. *Rhizopus microsporus* var. *azygosporus* CBS 357.93 and *R. microsporus* var. *microsporus* ATCC 52813 had the smallest genome sizes at 16 and 26 Mb respectively (Figure 3). Surprisingly, the assembled genome sizes varied nearly threefold between *R. microsporus* strains, with the largest assembled genomes in strains B9645 (∼66 Mb) and B9738 (∼75 Mb). The two other *R. microsporus* genomes (strains M201021 and B7455) are comparable to the average genome size of 44.5 Mb observed in the *R. stolonifer* and *R. arrhizus/delemar* clades.

### Role of transposons in genome size and structure

All of the analyzed genomes contain more than 40% repetitive sequences (Figure 3). However, only ∼10% of those repeats are likely to be active transposons with intact transposase-coding regions; the remainder of the repetitive regions are composed of either simple repeats or remnants of ancient transposons. The repetitive content also correlates with the assembly quality. The best assembled genome of *R. delemar* contains the largest number of transposons and the greatest fraction of genome content occupied by repetitive sequences.

The GC content of the *Rhizopus* genomes ranges from 32.5% for *R. microsporus* B9645 to 37.5% for *R. microsporus* 52813. The GC content can have dual influential roles in transposon biology: on one hand, AT-rich regions are favored as transposition sites, but on the other hand, the GC content is influenced by the mobile elements themselves. Transposons tend to insert into transposon-rich regions, possibly producing a genomic niche for the acquisition of additional elements.

**Figure 4:**
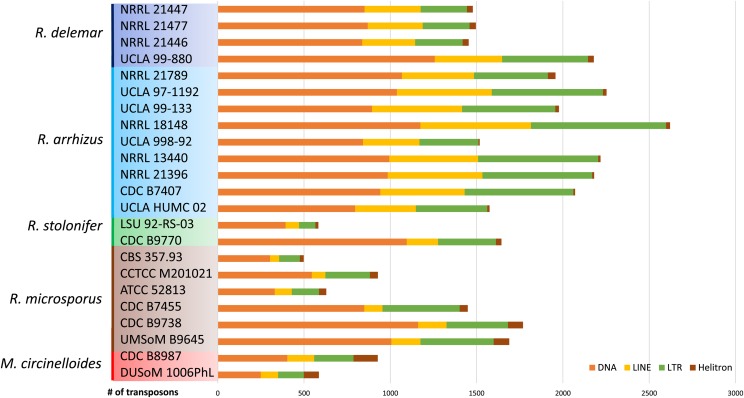
Number of transposons with ORFs typical of LTR/LINE/DNA/Helitron elements. Colored boxes are used to highlight the species identity of each strain.

Mucorales do not seem to have efficient or deployable genome defense mechanisms against transposable elements; some Mucorales species appear to have rampant transposon proliferation. The most widespread elements are from the LINE (L1 and RTE) and LTR retrotransposon (Ty3/Gypsy) families, which are prevalent in most eukaryotic genomes. *Rhizopus* genomes harbor 12 to 165 copies of DIRS elements with a YR transposase and only single cases of Ty1/Copia elements. DNA transposons with DDE transposases from the super-families Mutator-like, Merlin, PIF-Harbinger, and Tc1/Mariner are present in all genomes. Notably, remnants of Caulimovirus sequences with *pol* fragments are present in one-third of the analyzed genomes (Figure 4, Table S3).

### Mating type locus variation among Rhizopus species

**Figure 5:**
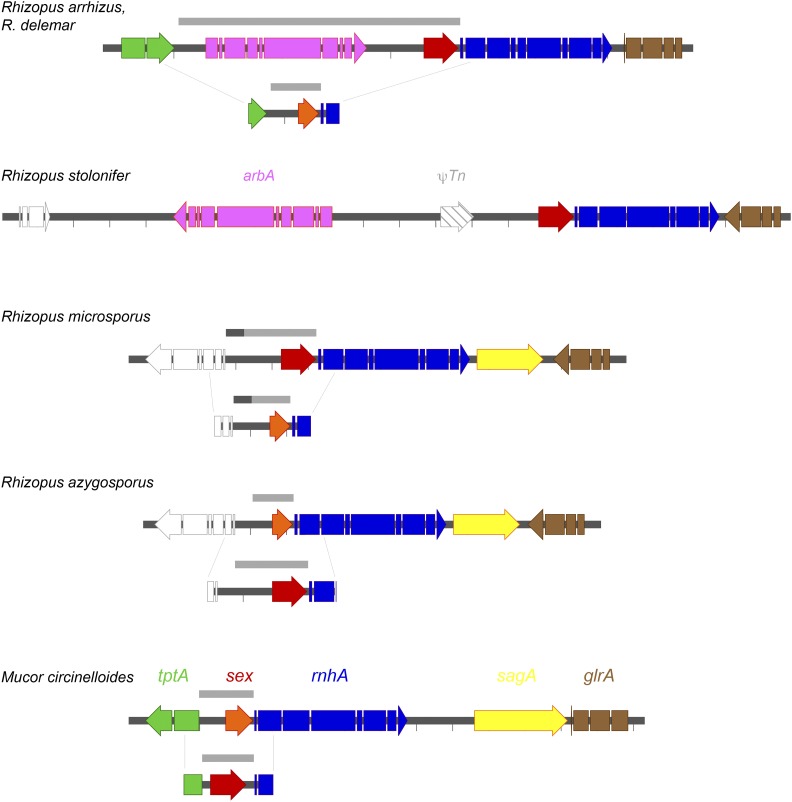
The structure of the mating type (*sex*) loci in representative strains of the four *Rhizopus* species and in the outgroup *Mucor circinelloides* (NCBI sequence accession numbers are HQ450311-12, HQ450315-16 (*R. arrhizus*), HQ450313 (*R. delemar*), MG967658 (*R. stolonifer*), MG967659-60 (*R. microsporus* var. *azygosporus*), HM565940-41 (*M. circinelloides*). Note that the structure of the mating type locus is shown for *R. arrhizus* and *R. delemar* together; these two closely related species share a similar arrangement in the mating type locus. The color-coding for each gene is listed above the *M. circinelloides* homologs, except for *arbA* (which is listed above the *R. stolonifer* graphic). Red arrows indicate *sexP* and orange arrows indicate *sexM* genes. Genes depicted in white are genes that were not previously found physically linked with the sex loci in Mucorales species. The gray bars above the diagrams indicate the idiomorphic regions that differ between (+) and (–) strains. Genome sequence is available for only a (+) strain of *R. stolonifer*, so the extent of the idiomorphic region, and the nature of the (–) form are unknown. There is a remnant of a transposable element (ψTn) between the *arbA* and *sexP* genes in *R. stolonifer*. For *R. azygosporus*, both *sexM* and *sexP* idiomorphic sequences are found in the same strain. Dashes indicate spacing of 1 kb.

The mating type or *sex* loci of heterothallic mucoralean fungi are defined as either (+) or (-) based on a *sex* gene that encodes a HMG domain-containing protein, flanked by an RNA helicase (*rnhA*) on one side and a triose phosphate transporter (*tptA*) on the other (Schulz *et al.* 2016, Lee and Idnurm 2017). However, deviations from this composition are observed in all *Rhizopus* genomes. For example, in *R. arrhizus* and *R. stolonifer*, a large gene with a BTB domain (contained in BR-C, *ttk* and *bab* genes) flanks the *sex* gene opposite *rnhA*, and the (+) and (-) *R. microsporus* strains and (+) *R. stolonifer* lack a flanking *tptA*. BLAST searches of the *R. stolonifer* (-) genome identified a mating locus that is structured similarly to those in (-) strains of *R. arrhizus* except that the *sex* gene of *R. stolonifer* does not have a *tptA* homolog adjacent to it. The resolution of the sequence for a (+) isolate of *R. stolonifer* is currently too low to resolve the *sex* locus in this species (Figure 5). Adjacent to the *R. microsporus sex* locus are genes encoding transcription factors (*sagA*) and a glutathione reductase (*glrA*), which are located adjacent to the *sex* locus in other mucoralean fungi (Idnurm 2011, Schulz *et al.* 2016). In most species, the edges between the conserved sequences on either side of the divergent *sex* locus are generally clearly defined. *Rhizopus microsporus* var. *azygosporus* contains two *sex* loci. Alignment of each against the other reveals the equivalent of idiomorphic regions carrying either *sexM* or *sexP* genes. However, in the case of *R. microsporus*, there is an additional of approximately 500 bp region (dashed gray in Figure 5), where the identity between the two mating types is 92%.

## Discussion

### Inadequacy of single-gene phylogenies to resolve relationships within Rhizopus

The genus *Rhizopus* has been traditionally divided into three large clades based on spore size: sporangiospores ranging in diameter from 6.5 µm in *R. microsporus*, 8 to 10 µm in *R. arrhizus*, and up to 13 µm in *R. stolonifer* (Schipper 1984, Zheng *et al.* 2007). This division is also supported by our phylogenetic tree based on non-molecular characters (Figure 2B). Such a division lends itself to simple microscopic identification, especially for the most commonly found species in the genus.

However, different single-gene phylogenies have produced sharply contrasting results. A phylogeny built with ITS sequences places *R. stolonifer* as sister to the remainder of the Rhizopodaceae, with *R. arrhizus*, *R. delemar* and *R. microsporus* forming a single clade. Additionally, this ITS phylogeny groups the genera *Syzygites* and *Sporodiniella* within *Rhizopus* in a sister relationship to *R. stolonifer* (Walther *et al.* 2013). Phylogenies built using other rDNA loci (*e.g.*, 18S and 28S) also place *R. stolonifer* at a basal position, while *R. microsporus* occupies the most distant branch of the tree (Abe *et al.* 2006). Similar results were obtained by Liou *et al.* (Liou *et al.* 2007) using the 28S D1-D2 region of rDNA for 34 strains of the genus *Rhizopus*, and by Voigt *et al.* (Voigt *et al.* 1999) using 18S sequences of six *Rhizopus* strains. The clade that includes *R. arrhizus* and *R. delemar* in these single-gene phylogenies is placed either independent of the *R. microsporus* and *R. stolonifer* clades, or in close relationship to the *R. stolonifer* complex (Abe *et al.* 2006). In the trees produced by Abe *et al.* (Abe *et al.* 2010) using a greater number of strains and genes (ITS, *actin-1* and *EF-1α*), the trees were generally congruent with those described previously. However, the elongation factor 1-alpha (*EF-1α*) phylogeny placed the *R. stolonifer* group between the *R. microsporus* and *R. arrhizus/delemar* clades. Quite different results were obtained by Liu *et al.* (Liu *et al.* 2007), who used ITS and *pyrG* phylogenetic trees for 23 *Rhizopus* isolates, placing the *R. microsporus* clade at the base of the genus while *R. arrhizus/delemar* formed the most distant clade.

The inconsistencies in topologies vary depending on which genes are analyzed or which phylogenetic methods are used. The discrepancies demonstrate that single-gene approaches are of limited value in generating a phylogeny that robustly resolves members of the genus *Rhizopus*. The use of multiple genes (actin, *EF-1α*, 18S, and 28S rDNA) within the broader phylogenetic context of the entire Mucorales places *R. stolonifer* as the most distant clade within *Rhizopus*, together with *Sporodiniella* and *Syzygites*, although *R. microsporus* was placed sister to the rest of the Rhizopodaceae family (Hoffmann *et al.* 2013).

Here, using 192 orthologous protein-coding genes derived from whole-genome sequencing of representative species of the genus *Rhizopus*, we obtained a robust and well-supported phylogeny for the genus. The tree topology supports the findings of Liu *et al.* (Liu *et al.* 2007) and suggests that *R. microsporus* is a monophyletic clade sister to other *Rhizopus* clades (Dolatabadi *et al.* 2014), while *R. stolonifer* is sister to *R. arrhizus* and *R. delemar*. All four species are monophyletic, although *R. arrhizus* and *R. delemar* are closely related and are not differentiated based on morphology (Gryganskyi *et al.* 2010). Our tree topology is also congruent with that obtained by Chibucos *et al.* (Chibucos *et al.* 2016) using 76 orthologous proteins from the genomes of 16 *Rhizopus* strains. Our results are further supported by a non-molecular phylogenetic tree that was built using 14 morphological and two ecological characters. Including genome data of other *Rhizopus* species in future analyses might alter the status of some species. We suspect that in the future there will be a reduction in the number of accepted species since some of these taxa may actually be phylogenetically nested within *R. arrhizus*, *R. delemar*, *R. stolonifer* or *R. microsporus*. There is some early evidence of this pattern; rDNA data suggest that *R. sexualis* is likely part of the *R. stolonifer* clade (Abe *et al.* 2006). However, other species delimiting criteria can be applied to some species which exhibit homothallic life cycle (*R. homothallicus*, *R. sexualis*) compared to the rest of the species which are known to be heterothallic.

### Transposons as agents that impact genome size

Most genomes of *Rhizopus* species contain numerous simple sequence repeats (Figure 3) and have a genome size of ∼45 Mb. This is relatively large compared to other fungi, although most of the available genomes represent species in the Dikarya (Ascomycota and Basidiomycota) (Stajich 2017).

Larger genomes generally harbor more mobile elements (Elliott and Gregory 2015), and genome inflation may be due to incomplete elimination of transposons arising from whole genome duplication and/or inefficient or weakened genome defense mechanisms, which has been observed in other taxa (Chuong *et al.* 2017). The genome composition of DNA transposons, LTR retrotransposons, and LINE retrotransposons is typical of most fungi and similar to other Mucorales (Muszewska *et al.* 2011). LTR retrotransposons and Tc1/Mariners have been described as the most abundant transposons in *R. delemar* (Ma *et al.* 2009). Transposon proliferation may have occurred alongside whole-genome duplication (WGD) events, or transposon proliferation may even be a mechanism that influences genome duplication (Ma *et al.* 2009, Carbone *et al.* 2014).

### Additional evidence for a common genome duplication in the Mucorales

Genome size correlates with the number of chromosomes. Only a handful of studies from a single Korean research group have explored *Rhizopus* karyotypes. They reported a wide range of chromosome numbers, from six in *R. oligosporus* (=*R. microsporus*) to 16 in *R. nigricans* (=*R. stolonifer*). However, these studies, which are more than three decades old, reveal conflicts even between different isolates of the same species (synonyms) (Min 1984, Flanagan 1969, Ganguly and Prasad 1971). Based on the data we present here, the higher chromosome count might be consistent with whole-genome expansion events.

Mapping the genome size onto our phylogenetic tree (Figure 2) suggests that smaller ancestral *Rhizopus* genomes expanded twofold in several branches, possibly through incomplete duplication, hybridization, or other mechanisms of genome expansion. This size variation is consistent with the occurrence of multiple genome duplication events during the evolution of species within the genus *Rhizopus* (Ma *et al.* 2009). Striking evidence for duplication events is present in *R. microsporus*, with genomes of double (49 Mb) or even nearly triple the size (65 to 75 Mb) of the smallest genome sequenced in this study. The genome sizes of other clades of the genus *Rhizopus* (*R. arrhizus*, *R. delemar*, and *R. stolonifer*) are larger (45 Mb on average) but also more uniform compared with the *R. microsporus* clade. One of the possible reasons for a larger genome size and potential evidence of genome duplication or triplication could also be hybridization between different species of this genus as observed by Schipper *et al.* (Schipper *et al.* 1985). Evidence of genome duplications—both recently and in the past—in *Rhizopus* mirrors previous observations from analysis of other mucoralean lineages (Corrochano *et al.* 2016). However, obtained data on genome size, sequencing coverage and the number of genes and transposons should be treated with caution. The quality of genomic DNA, the sequencing technology, and the genome assembly methods all have a large impact on the final genome. These technical, non-biological factors could be important and might account for the differences in genome sizes in some of the clades. For example, the genomes of *R. arrhizus* average 42.4 Mb in size but deviate by ±5 Mb between samples with no evidence of genome duplication. Similar deviation occurs within the other clades suggesting that additional genomes will help to identify the sources of variation between genomes in the same clades.

### Structure of the mating type/sex locus

All of the *Rhizopus* genomes we examined contain a clear *sex* gene cluster (Idnurm *et al.* 2008, Gryganskyi *et al.* 2010, Lee and Heitman 2014). The structure of the *sex* locus and the relationship of the surrounding genes are not fully understood in Mucorales, especially with the increasing number of Mucorales genome sequences becoming available. The presence of the *glrA* homolog (which encodes a putative glutathione reductase) instead of the *tptA* gene in both *R. microsporus* (+) and (-) strains, in close proximity to *rnhA*, is also observed in the closely related homothallic taxon *Syzygites megalocarpus*. RNA helicase (*rnhA*) mediates RNAi-dependent epimutational silencing in *Mucor circinelloides* (Calo *et al.* 2017). Thus, it can be inferred that *R. microsporus* has maintained the ancestral structure of the mating locus that is common to other mucoralean fungi.

Our data suggest that the structure of the mating locus is flexible, even within a single genus, and that the arrangement of the gene triplet *tptA* – *sexP*/*sexM* – *rnhA* is not universally conserved among Mucorales species. We did not identify a *tptA* gene in *R. stolonifer* (+) strains, but rather a predicted protein-coding gene (*arbA*) containing a BTB domain. The same gene configuration has been found in both *R. arrhizus* and *R. delemar* (Gryganskyi *et al.* 2010). In addition, the intermediate regions between the genes in this cluster in *R. stolonifer* are much larger than in *sex* loci of other mucoralean fungi, and the *arbA* gene is reversed in orientation compared to *R. arrhizus* (+) strains. The finding of the unusual mating locus structure in *R. stolonifer* (+) could be an artifact of the genome assembly process.

The *R. microsporus* var. *azygosporus* strain CBS 357.93 had low sequence coverage and resulted in a poor genome assembly of just 16 Mb. Nonetheless, sufficient information was gained to characterize the *sex* genes in this strain and to show that it carries homologs of both *sexM* and *sexP*. *Rhizopus azygosporus* was described as a new species based on its formation of azygospores, a zygospore-like cell that forms in a parthenogenic manner without the fusion between two “gametes” (Yuan and Jong 1984). Subsequent analyses revealed strong similarities to *R. microsporus*, and hence its reduction to a varietal status (Schwertz *et al.* 1997, Zheng *et al.* 2007). The presence of both transcriptional regulators (which distinguish the two mating types or sexes in heterothallic species) within a single strain is one mechanism that leads to homothallism in fungi. Whether CBS 357.93 represents an unreduced fusion event between (+) and (-) strains of *R. microsporus* or a true example of homothallism is not yet clear. Improved genome sequencing of CBS 357.93 and other *R. azygosporus* strains may help to clarify. Although more data are needed, evidence from this strain suggests that one mechanism by which genome duplication could occur in the Mucorales is through the fusion of strains of opposite sex.

### A new understanding of the evolution of Rhizopus

*Rhizopus* is an enigmatic genus comprising species that are ubiquitously found in nature and that play important roles in agriculture, industry, and human health. Despite the widespread prevalence of *Rhizopus*, understanding the evolution of species within the genus has remained challenging. Our study used a genome-wide phylogenomic approach to provide robust resolution of species within *Rhizopus*. The included *Rhizopus* genomes separated into three major clades with significant bootstrap support: *R. microsporus*, *R. stolonifer*, and a clade containing the closely related species *R. arrhizus* and *R. delemar*. Strains from the *R. microsporus* clade have both the smallest and the largest genomes, ranging from 26 to 75 Mb, possibly caused by recurrent whole-genome duplication events and/or hybridization. Additional duplication events have given rise to two morphologically distinct yet closely related clades of *R. stolonifer* and *R. arrhizus* (including *R. delemar* or *R. arrhizus* var. *delemar*), the genomes of which underwent incomplete duplication, with an average size ranging from 38 to 48 Mb. However, in addition to duplication events, the number of transposable elements is also positively correlated with a larger genome size and can lead to genome inflation. A comparison of the mating type loci in these species showed a flexible architecture in which only two genes—*sex* and *rnhA*—are consistently adjacent to one another. A comprehensive sampling of all known species of the genus *Rhizopus* and two closely related genera, *Syzygites* and *Sporodiniella*, will further resolve lineage relationships and establish a comparative framework to continue studying the evolution of genome size and gene content in mucoralean fungi.
