# Corrigendum

**DOI:** 10.1534/g3.119.400465

**Published:** 2019-07-31

In the article by A. P. Gryganskyi, J. Golan, S. Dolatabadi, S. Mondo, S. Robb, A. Idnurm, A. Muszewska, K. Steczkiewicz, S. Masonjones, H.-L. Liao, M. T. Gajdeczka, F. Anike, A. Vuek, I. M. Anishchenko, K. Voigt, G. S. de Hoog, M. E. Smith, J. Heitman, R. Vilgalys, and J. E. Stajich (*G3: Genes|Genomes|Genetics* 8(6): 2007-2018) entitled “Phylogenetic and Phylogenomic Definition of *Rhizopus* Species,” the affiliation information included for S. Dolatabadi was incorrect in two instances. On page 2007, in the author affiliation section, “Sabzevar University of New Technologies” was corrected to “Sabzevar University of New Technology.” On pages 2007 and 2008, the previous address for S. Dolatabadi has been removed.

